# The Effects of Combining Web-Based eHealth With Telephone Nurse Case Management for Pediatric Asthma Control: A Randomized Controlled Trial

**DOI:** 10.2196/jmir.1964

**Published:** 2012-07-26

**Authors:** David Gustafson, Meg Wise, Abhik Bhattacharya, Alice Pulvermacher, Kathleen Shanovich, Brenda Phillips, Erik Lehman, Vernon Chinchilli, Robert Hawkins, Jee-Seon Kim

**Affiliations:** ^1^Center for Health Enhancement Systems StudiesUniversity of Wisconsin-MadisonMadison, WIUnited States; ^2^Blue Health IntelligenceChicago, ILUnited States; ^3^Department of PediatricsUniversity of Wisconsin-MadisonMadison, WIUnited States; ^4^Department of Public Health SciencesPenn State Hershey College of MedicineHershey, PAUnited States; ^5^Department of Journalism and Mass CommunicationUniversity of Wisconsin-MadisonMadison, WIUnited States; ^6^Department of Educational PsychologyUniversity of Wisconsin-MadisonMadison, WIUnited States

**Keywords:** Asthma, asthma information, childhood disease, case management, patient education, eHealth, social support

## Abstract

**Background:**

Asthma is the most common pediatric illness in the United States, burdening low-income and minority families disproportionately and contributing to high health care costs. Clinic-based asthma education and telephone case management have had mixed results on asthma control, as have eHealth programs and online games.

**Objectives:**

To test the effects of (1) CHESS+CM, a system for parents and children ages 4–12 years with poorly controlled asthma, on asthma control and medication adherence, and (2) competence, self-efficacy, and social support as mediators. CHESS+CM included a fully automated eHealth component (Comprehensive Health Enhancement Support System [CHESS]) plus monthly nurse case management (CM) via phone. CHESS, based on self-determination theory, was designed to improve competence, social support, and intrinsic motivation of parents and children.

**Methods:**

We identified eligible parent–child dyads from files of managed care organizations in Madison and Milwaukee, Wisconsin, USA, sent them recruitment letters, and randomly assigned them (unblinded) to a control group of treatment as usual plus asthma information or to CHESS+CM. Asthma control was measured by the Asthma Control Questionnaire (ACQ) and self-reported symptom-free days. Medication adherence was a composite of pharmacy refill data and medication taking. Social support, information competence, and self-efficacy were self-assessed in questionnaires. All data were collected at 0, 3, 6, 9, and 12 months. Asthma diaries kept during a 3-week run-in period before randomization provided baseline data.

**Results:**

Of 305 parent–child dyads enrolled, 301 were randomly assigned, 153 to the control group and 148 to CHESS+CM. Most parents were female (283/301, 94%), African American (150/301, 49.8%), and had a low income as indicated by child’s Medicaid status (154/301, 51.2%); 146 (48.5%) were single and 96 of 301 (31.9%) had a high school education or less. Completion rates were 127 of 153 control group dyads (83.0%) and 132 of 148 CHESS+CM group dyads (89.2%). CHESS+CM group children had significantly better asthma control on the ACQ (*d *= –0.31, 95% confidence limits [CL] –0.56, –0.06, *P *= .011), but not as measured by symptom-free days (*d *= 0.18, 95% CL –0.88, 1.60, *P *= 1.00). The composite adherence scores did not differ significantly between groups (*d *= 1.48%, 95% CL –8.15, 11.11, *P *= .76). Social support was a significant mediator for CHESS+CM’s effect on asthma control (alpha = .200, *P *= .01; beta = .210, *P *= .03). Self-efficacy was not significant (alpha = .080, *P *= .14; beta = .476, *P *= .01); neither was information competence (alpha = .079, *P *= .09; beta = .063, *P *= .64).

**Conclusions:**

Integrating telephone case management with eHealth benefited pediatric asthma control, though not medication adherence. Improved methods of measuring medication adherence are needed. Social support appears to be more effective than information in improving pediatric asthma control.

**Trial Registration:**

Clinicaltrials.gov NCT00214383; http://clinicaltrials.gov/ct2/show/NCT00214383 (Archived by WebCite at http://www.webcitation.org/68OVwqMPz)

## Introduction

Asthma and other chronic diseases pose a great risk as the United States attempts to decrease its health care costs. Research suggests that the active ingredients of chronic disease management include long duration [1,2], assertive outreach [3], monitoring [4-6], prompts [7-9], action planning [10-12], case management [13-15], and peer [16-18] and family [19-21] support. All of these can potentially be provided by information and computer-based technologies and sensors.

Poor control of pediatric asthma affects low-income and minority children disproportionately and contributes to more than 14 million lost school days and 3 million lost parental workdays per year [22]. Daily controller medications can manage even severe asthma [23], but adherence is low, especially for the underserved [24]. Asthma education programs, with their low participation rates, have had mixed results [23]. Nurse case management can reduce asthma-related emergency care and hospitalization costs, but it is expensive [25,26]. Child-focused, Web-based asthma education and games (eHealth) have improved knowledge, asthma control, and medication adherence in the short run [27-29]. However, parents tend to overestimate their child’s medication-taking skills and actual adherence [30]. This suggests that parental involvement might be beneficial for managing pediatric asthma. Integrating phone-based clinician care into asthma eHealth programs for adults has shown significant promise in behavioral and asthma outcomes [31]. Interventions such as CHESS+CM, based on self-determination theory [32] and self-efficacy [33], provide information, social support, and skill-building tools for self-managing the disease. These interventions, which aim to increase confidence, competence, relatedness, and autonomous motivation, have been used successfully in asthma education programs [28,29,32,34]. However, the factors associated with these theories have not been tested for their mediational effects on adherence to controller medications or asthma control. Understanding this is important for developing an asthma eHealth system that balances the various functions—information, social support, and skill building—to the best effect for children and their parents.

We, therefore, hypothesized that a parent-focused intervention that integrates monthly telephone nurse case management with a comprehensive, interactive asthma eHealth program could improve asthma control and medication adherence. We surmised that these effects would be mediated by social support, self-efficacy, and asthma information competence. This paper reports the results of a randomized controlled trial funded by the US National Institute of Nursing Research.

## Methods

### Intervention

The year-long intervention called CHESS+CM consisted of an eHealth program, Comprehensive Health Enhancement Support System (CHESS), and a monthly telephone call to the parent from an asthma nurse case manager (CM). CHESS is an umbrella name for several eHealth systems developed and tested for the past 25 years at the University of Wisconsin-Madison. CHESS modules provide information, adherence strategies, decision-making tools, and support services in attractive, easy-to-use formats. The most important strength of CHESS modules may be the closed, guided universe of tailored information and support in an integrated package with efficient navigation, eliminating the need for complicated search and discovery. In randomized efficacy trials, CHESS modules significantly improved quality of life and self-efficacy for women with breast cancer versus control and Internet groups, and quality of life and costs of care in people infected with the human immunodeficiency virus [35,36]. CHESS programs also have demonstrated the effectiveness of using self-determination and self-efficacy theories to improve information competence, health care participation, and social support among cancer patients [35,37]. The CHESS module used in this study was designed specifically for asthma.

The project was carried out with a University of Wisconsin-based team of educators, pharmacists, and nurse practitioners specializing in asthma rather than with staff from the five managed care organizations (MCOs) from which participants were recruited. University of Wisconsin-based nurse practitioners also monitored the progress of recruitment. The project director monitored the discussion group within CHESS to ensure that calls for help were rapidly addressed and that inaccurate information was not shared. The full trial protocol is available at http://www.webcitation.org/69E2cXZbo.

**Figure 1:**
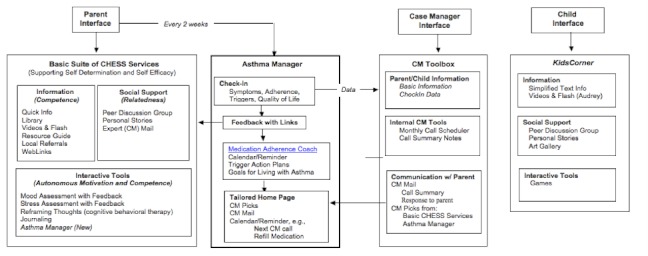
Comprehensive Health Enhancement Support System (CHESS) services for parent, case manager, and child. CM = case management.

As Figure 1 shows, CHESS for asthma had three audiences: parents, children, and case managers. Parents received comprehensive information based on the National Asthma Education and Prevention Program guidelines [21,22,38], a peer discussion group, case manager email, and the Asthma Coach, which assesses the child’s asthma and the parent’s and child’s well-being. CHESS provided tailored feedback and links to salient CHESS content and other interactive tools. Children received simplified information in game and audiovisual formats, as well as social support via a peer discussion group and personal stories. No major bug fixes or downtimes occurred. Asthma-related content was updated annually over the course of the study. Otherwise, no major modifications were made to the system. No significant secular events took place during the study period.

**Figure 2:**
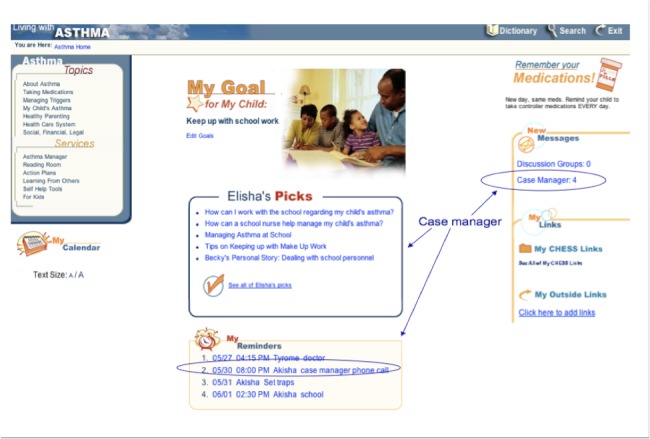
Parent’s home page of the Comprehensive Health Enhancement Support System (CHESS) asthma module.

The case manager received tools to schedule monthly phone calls with the parent, view parents’ Asthma Coach entries, enter phone call notes, and send and receive case management mail to and from the parent, as well as a “prescription pad” to place CHESS resources on the parent’s home page [21]. Monthly case management calls to the parent assessed the child’s asthma, medication adherence, and psychosocial challenges, and provided relevant education and support. On completing a call, the case manager entered notes in the case management toolbox and then sent the parent a summary via case manager email with links to salient CHESS resources, which appeared on the parents’ CHESS home page, as shown in Figure 2 [21]. These features were designed with user input for content and usability. For a more complete description of the CHESS asthma module and its development, see Wise et al [21]. The module is available at https://chess.wisc.edu/asthmamobile/. The code name is *uwmadison *and the password is *testing*. Screenshots of the program are available on request.

### Invitation and Recruitment

Eligible participants were parents or other adults functioning as parents, such as grandmothers, who were able to read at a sixth-grade level and had children ages 4–12 years with a diagnosis of asthma (*International Classification of Diseases*, 9th revision code 493) or wheezing (code 786.07); a prescribed asthma controller medication; and poor medication adherence, defined as having missed more than one medication refill or having an emergency department visit or hospitalization because of poor asthma control. Originally children were identified through the health care utilization and pharmacy claim databases at four MCOs (MCOs 1–4) and the Wisconsin Medicaid Program from one urban–rural county (Dane County, which is also the home of the University of Wisconsin-Madison) and seven surrounding rural counties (Columbia, Dodge, Green, Iowa, Jefferson, Rock, and Sauk). MCO 5 in Milwaukee was added after it became clear that MCOs 1–4 could not produce enough participants with poorly controlled asthma. MCO 5 served an entirely Medicaid population in Milwaukee County and had the state’s highest rates of asthma-related emergency department visits and overnight hospital stays [39].

All research materials and procedures were approved by the University of Wisconsin’s Health Sciences Review Board, as well as by the ethics and review boards for each of the five MCOs. Recruitment was initiated by a letter from the MCO or Wisconsin Medicaid Program to parents of study-eligible patients with an opt-in or an opt-out card regarding a phone call from the study nurse, depending on the organization’s institutional review board policies [40]. Recruiters screened for eligibility, described the study (as a comparison of two approaches to asthma control) and its risks and benefits, and scheduled an intake interview for people who agreed to participate.

The study had four key risks. (1) The key risk for participants was the possibility of their replacing effective medical care with unproven treatments. To reduce this risk, each survey asked whether parts of the child’s medical treatment had been abandoned against medical advice. In addition, we scanned discussion group entries in CHESS. If we found indications of abandonment, we contacted parents to express our concern about potential risks. (2) Participants’ misinterpreting information in CHESS posed another risk. The information was presented at a sixth-grade reading level and screened by asthma experts to reduce this risk. Disclaimers also cautioned that the computer is not a substitute for seeking medical attention and that comments in the social media may not be factual. (3) To reduce the risk of anonymity being breached, participants were assigned a blind code number. All data had names removed and code numbers attached. (4) Participants’ divulging confidential information was another risk in the study. We frequently warned users about this, and we used digital signatures to warn users if CHESS was altered.

### Enrollment

Parents and children participated in a prerandomization intake appointment with a study case manager at asthma clinics associated with MCOs 1–4 and, in MCO 5, at a community center. The study team traveled twice a month to the community center to conduct intakes after school and into the early evening [40]. At all locations, childcare, snacks, and transportation were provided as needed. Parents were asked to bring the child’s medications to the intake interviews. Intakes lasted 45–90 minutes. Potential participants were informed of (1) the nature and purpose of the study, (2) the financial compensation offered (US $35 for completing each of four surveys and providing 2 spirometry readings), (3) the types of data to be collected from claims records, surveys, and computer-use tracking, (4) the intervention to be given to the experimental group, (5) the nature and reasons for random assignment, (6) the measures used to insure the confidentiality of data collected, (7) the timeline of the study, (8) the duration of the study (12 months, to capture the seasonality of asthma), and (9) the University of Wisconsin-Madison being the research organization for the project. Consent was documented by obtaining signed, institutional review board-approved consent forms containing all of the above information. The consent forms were kept in a locked file at CHESS. Intake appointments assessed parental ability to read at a sixth-grade level by asking parents to read aloud the consent letter. The appointments also included recording medications and doses, a spirometry test, and the child’s asthma history. A researcher administered a pretest survey with training on completing the asthma diary and provided individualized asthma education as needed. Regardless of study arm, case managers notified MCO staff about children with uncontrolled asthma for further evaluation.

### Randomization

Researchers at the University of Wisconsin generated the random allocation sequence. Nurses conducting consent, assent, and pretests were given sequentially numbered envelopes containing the random assignment for each participants. It was not possible to blind participants or outcome assessors. We did blind those analyzing the data.

Participants were equally randomized according to their MCO and then blocked by severity and by Medicaid status. Randomization occurred upon receipt of the run in diaries for MCO 1-4 subject and after just the intake for MCO 5 participants [40]. The CHESS+CM group received a 45-minute training session. Laptop computers, land phone lines, and Internet service were provided, as needed. MCOs 1–4 and Wisconsin Medicaid Program participants received one-on-one training at home on an Internet-enabled computer with the live CHESS program. MCO 5 participants received group training at the community center where they had had their intake interview. Because that center lacked Internet access and most participants borrowed study laptops, training on using the laptop and CHESS was guided by an interactive compact disc. All users were instructed to use CHESS whenever they wished. No minimum expectations were placed on users.

All participants, regardless of study condition, received a call from the project manager 1 week after randomization to see how things were going. They also received with their mailed surveys at 3, 6, 9, and 12 months a packet of educational materials about asthma control, child development, parenting, and community resources. Parents and children returned to the clinic or community center for an exit interview that included taking the same measures used at the intake appointment. Exit interviews were conducted at home for 40 difficult-to-reach MCO 5 parents.

### Measures

Asthma control was measured via two self-reports collected at baseline and at 3, 6, 9, and 12 months. Juniper and colleagues’ Asthma Control Questionnaire (ACQ) was administered orally at the intake and exit interviews and self-administered via mailed surveys at the intervening time points [41]. This well-validated, 6-item, 7-point Likert scale measured daytime and nocturnal symptoms, missed school, and rescue medication use over the previous 7 days [41]. Lower scores indicated better asthma control. Scales were created as a mean of all items. Surveys with missing ACQ items were not calculated and counted as missing data. Symptom-free days were calculated from 2-week diaries as the absence of asthma-related nighttime awakenings, daytime symptoms, bronchodilator use, unscheduled physician visits, or school absences [42]. The study included a run-in period of 3 weeks before randomization during which participants kept asthma diaries that were used to collect baseline data on symptom-free days.

Adherence to asthma controller medications was measured as the mean composite score from self-reported 2-week asthma diaries at baseline and 3, 6, 9, and 12 months [42]. The ratio of medication to possession was calculated from MCO and Wisconsin Medicaid Program pharmacy claims data as the actual versus expected prescription refill rate for each participant’s prescription.

Social support, self-efficacy, and information competence were adapted from 5-point Likert scales used successfully in prior CHESS studies [35]. The 6-item social-support scale assessed the availability and reliability of social, emotional, and instrumental support. The 6-item self-efficacy scale assessed asthma problem-solving skills and strategies, along with perceived competence, goal attainment, and comparison with others’ skills. The 4-item information competence scale assessed parents’ understanding of asthma information needs, difficulty in obtaining such information, satisfaction with their strategies, and level of control using the information. Scales were created as a mean of all items. If more than 1 item was missing, the scale score was not computed.

### Statistical Analysis

Our study was powered based on an expected 320 dyads completing the trial; 259 completed the study. We anticipated a dropout rate of 20% but had a rate of 14% (42/301).

Descriptive statistics established baseline characteristics for the CHESS+CM and control groups and for participants with missing data. We used 1-way analysis of variance to compare differences between the CHESS+CM and control groups and between dropouts and those who completed the study. For missing 3-, 6-, 9-, and 12-month posttest data, we used a general linear model procedure to test for equality of mean scores meeting the baseline criteria followed by a 1-way analysis of variance to determine the *P *value for the general linear model. Scores for outcome variables with no significant mean differences between 3, 6, 9, and 12 months were averaged to create a score for the entire intervention time [43].

Main outcomes were an intent-to-treat analysis with a repeated-measures, mixed model to account for the correlation between the five time points within participants, and to analyze the differences between the time points and baseline within and between the control and CHESS+CM groups. A Bonferroni adjustment for multiple comparisons yielded adjusted *P *values and confidence limits for mean estimates within each set of comparisons at the four time periods. Finding no significant differences, we averaged participants’ 3-, 6-, 9-, and 12-month data to create a score for the whole intervention time following the procedure described above. Comparisons of the change from baseline were made over the average of all posttest time points within the CHESS+CM group and the control group and between the two groups.

A multiple regression model, as described by MacKinnon [44], was used to analyze mediators for outcome variables that showed significant difference between the CHESS+CM and control groups.

Step 1 determined significant covariates by loading candidate variables for their premediated effects of the intervention tau on the outcome variable. Covariates included the outcome variable’s pretest score, Medicaid status, age, time since diagnosis, and asthma severity, as well as the parent’s age and education level and whether help with parenting was available from another adult. Covariates in step 2 included the pretest scores for the outcome and mediator variables and any significant covariate.

Step 2 used a multiple regression model for each mediator, whereby alpha = CHESS+CM’s effect on the mediator, beta = mediator’s effect on the dependent variable, and tau^1 ^= CHESS+CM’s effect on the dependent variable after the mediational test.

## Results

### Recruitment and Enrollment

**Figure 3:**
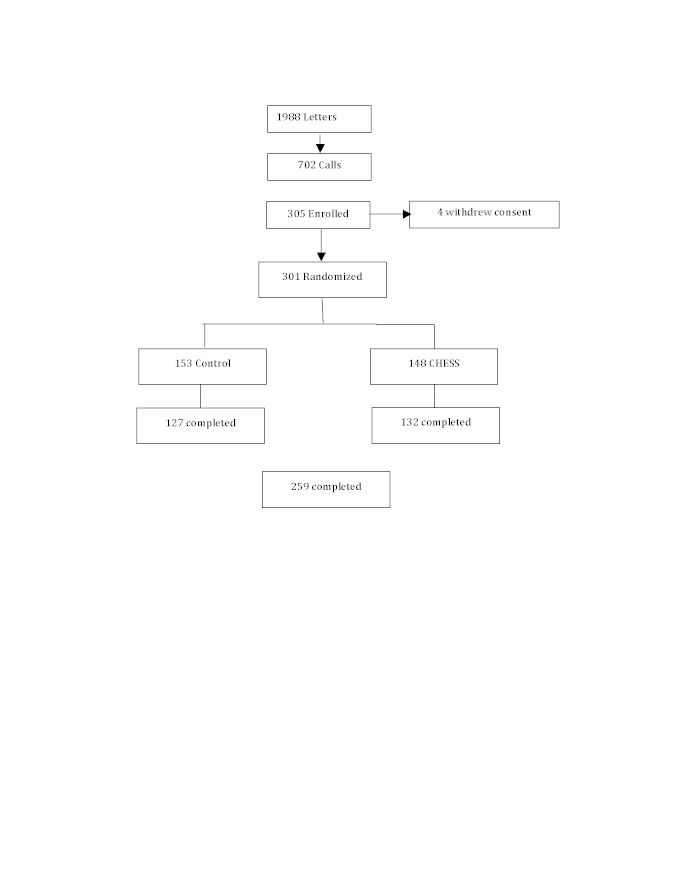
CONSORT diagram of sample pool, recruitment, and participation.

As Figure 3 shows, a total of 1988 invitation letters were mailed, 702 recruitment or eligibility screening calls were completed, and 305 parent–child dyads enrolled in the study. The enrollees accounted for 15.34% of the 1988 letters sent and 43.5% of the 702 completed phone calls. Data were collected from August 2, 2004 through August 16, 2007.

Reasons for nonenrollment were unable to be reached by phone, not eligible, did not have moderate to severe asthma, and too busy. Up to 3 did not come for their scheduled intake appointments. A total of 4 dyads dropped out after the intake but before randomization; thus, 301 were randomly assigned: 153 to the control group and 148 to the CHESS+CM group. Finally, 259 dyads (86.1%) completed the study. After randomization, 42 dropped out: 26 (17%) from the control group and 16 (11%) from the CHESS+CM group. The between-group dropout rate was not significant (*P *= .12). However, participants who dropped out were significantly less likely to be white or married, and more likely to be significantly younger, have lower asthma quality of life, and have less education. Children of dropouts had no significant differences in baseline ACQ scores, but had significantly lower pharmacy refill rates and more asthma-related school absences.

### Response Rates and Data Availability

Available data rates, shown in Table 1, were highest for self-reported data at baseline and 12 months, which involved direct interaction with a researcher. For example, the ACQ response rate was 98.7% at baseline and 82.7% at 12 months, but 58.1%, 52.8%, and 49.5% at the intervening time points. Missing pharmacy refill data, however, were highest at 12 months.

### Baseline Characteristics of Control and Intervention Groups

Table 2 shows no significant differences at baseline between the control and CHESS+CM groups for demographics, asthma status, Web use, or the mean outcome and mediator scores.

### Intervention Effects on Main Outcomes

Table 3 shows the mean difference in scores for the outcome variables between baseline and the mean scores measured at 3, 6, 9, and 12 months for the control group, the CHESS+CM group, and the difference between the CHESS+CM and the control group.

Symptom-free days as measured from asthma diaries improved significantly for the CHESS+CM group (odds ratio 1.38, *P *= .01) and less so for the control group (odds ratio 1.20, *P *= .29), but there were no significant between-group differences (odds ratio 0.18, *P *= 1.00). Asthma control as measured on the ACQ improved significantly for CHESS+CM (–0.42 on a 7-point Likert scale with lower scores indicating better control, *P *= .001) and not significantly for the control group (–0.11, *P *= .22). The between-group difference (–0.31) was significant (*P *= .01). The composite medication adherence score did not change significantly either within group or between the groups, with a 0.58% increase (*P *= .87) for the control group and 2.06% increase (*P *= .55) for the CHESS+CM group, and a 1.48% between-group difference (*P *= .76). Both groups reported declining adherence from diaries and had significant improvements in medication refills.

**Table 1:** Response rate and available data at study time points.

Measure		CHESS+CM^a^ (n = 148)	Control (n = 153)	Total (n = 301)
**Asthma Control Questionnaire**				
	Baseline	145 (98.0%)	152 (99.3%)	297 (98.7%)
	3 months	94 (64%)	81 (53%)	175 (58.1%)
	6 months	85 (57%)	74 (48%)	159 (52.8%)
	9 months	86 (58%)	63 (41%)	149 (49.5%)
	12 months	128 (86.5%)	121 (79.1%)	249 (82.7%)
**Diary data** ^b^				
	Baseline	128 (86.5%)	123 (80.4%)	251 (83.4%)
	3 months	91 (61%)	79 (52%)	170 (56.5%)
	6 months	84 (57%)	73 (48%)	157 (52.2%)
	9 months	84 (57%)	65 (42%)	149 (49.5%)
	12 months	108 (73.0%)	110 (72.9%)	218 (72.4%)
**Pharmacy claims data**				
	Baseline	103 (69.6%)	102 (66.7%)	205 (68.1%)
	3 months	64 (43%)	64 (42%)	128 (42.5%)
	6 months	78 (53%)	75 (49%)	153 (50.8%)
	9 months	70 (47%)	77 (50%)	155 (48.8%)
	12 months	59 (40%)	60 (39%)	119 (39.5%)

^a ^Comprehensive Health Enhancement Support System plus monthly nurse case management.

^b ^Diary data measured symptom-free days and self-reported medication adherence.

**Table 2:** Mean baseline values for demographics and main outcome and mediator measures.

Characteristic			Control (n = 153)	CHESS+CM^a^ (n = 148)	*P *value
Dropout, n (%)			26 (17%)	16 (11%)	.12
**Children’s characteristics**					
	Male gender, n (%)		87 (57%)	97 (66%)	.12
	Mean age (years), mean (SD)		8.18 (2.45)	7.65 (2.61)	.11
	Mean age at first asthma diagnosis (years), mean (SD)		3.16 (2.57)	2.79 (2.45)	.21
	African American, n (%)		84 (55%)	87 (59%)	.39
	Medicaid, n (%)		80 (52%)	74 (50%)	.69
	Yes to: n (%)				
		Prednisone at least once^b^	96 (63%)	101 (68%)	.33
		Hospital stays for asthma^b^	28 (18%)	22 (15%)	.51
		Emergency department for asthma^b^	78 (51%)	84 (57%)	.37
		Unplanned clinic visit^b^	116 (75.8%)	110 (74.3%)	.71
		Intensive care unit for asthma^b^	8 (5%)	4 (3%)	.39
		Asthma specialist^b^	87 (57%)	84 (57%)	.89
		Asthma action plan	78 (51%)	87 (59%)	.22
**Parents’ characteristics**					
	Mean age (years), mean (SD)		37.94 (8.06)	38.03 (9.81)	.92
	Female gender, n (%)		145 (94.8%)	138 (93.2%)	.59
	African American, n (%)		72 (47%)	78 (53%)	.25
	Marital status: with partner, n (%)		73 (48%)	73 (49%)	.82
	Highest level of education: high school or less		49 (32%)	47 (32%)	.67
**Outcome variables**					
	ACQ^c^, mean (SD) score		2.32 (1.11)	2.49 (1.26)	.21
	Symptom-free days, odds ratio (SD)		0.45 (0.39)	0.47 (0.38)	.75
	Composite adherence score, mean (SD) %		73.54 (47.81)	69.80 (26.96)	.43
	Pharmacy refill possession ratio, mean (SD)		56.86 (27.14)	58.44 (26.68)	.67
	Self-report 1, mean (SD) %		88.80 (51.4)	82.92 (27.09)	.30
	Self-report 2, mean (SD) %		89.97 (32.11)	87.10 (26.99)	.48
**Mediator variables**					
	Social support^d^, mean (SD) score		3.42 (0.73)	3.54 (0.71)	.13
	Self-efficacy^d^, mean (SD) score		3.58 (0.67)	3.67 (0.62)	.25
	Information competence^d^, mean (SD) score		3.13 (0.55)	3.25 (0.63)	.59

^a ^Comprehensive Health Enhancement Support System plus monthly nurse case management.

^b ^In the past year.

^c ^Asthma Control Questionnaire, response scale: 1 = excellent asthma control; 7 = very poor asthma control.

^d ^Response scale: 1–5.

**Table 3:** Intervention effects: baseline compared with mean of 3-, 6-, 9-, and 12-month scores.

Outcome			Within-control	Within-CHESS+CM^a^	CHESS+CM – Control
**Asthma control**					
	Symptom-free days odds ratio^b^				
		Difference	1.20	1.38	0.18
		95% CL^c^	0.98, 1.61	1.12, 1.71	-0.88, 1.60
		*P *value	.29	.01	1.00
	Asthma Control Questionnaire^d^				
		Difference^d^	–0.11	–0.42	–0.31
		95% CL	–0.29, 0.07	–0.60, –0.25	–0.56, –0.06
		*P *value	.22	.001^b^	.01
**Adherence**					
	Composite adherence scores^e^				
		Difference	0.58%	2.06%	1.48%
		95% CL	–6.24, 7.40	–4.74, 8.86	–8.15, 11.11
		*P *value	.87	.55	.76
	Pharmacy refill possession ratio				
		Difference	17.7%	13.76%	–3.95%
		95% CL	11.78, 23.62	7.83, 19.68	–12.33, 4.43
		*P *value	.001^b^	.001^b^	.35
	Self-report controller 1: inhaled corticosteroid^b^				
		Difference	–13.42%	–1.78%	11.64%
		95% CL	–21.49, –5.35	–18.67, –2.88	–8.65, 13.93
		*P *value	.001^b^	.008	.65
	Self-report controller 2: anticholingerics^b^				
		Difference	–1.85%	0.95%	2.81%
		95% CL	–12.05, 8.34	–8.73, 1.64	–11.26, 16.87
		*P *value	.72	.85	.69

^a ^Comprehensive Health Enhancement Support System plus monthly nurse case management.

^b^ *P*< .01.

^c ^Confidence limits.

^d^ *P *≤ .001.

^e ^Sum of self-reported adherence data and pharmacy refill data.

### Mechanisms of CHESS+CM Effect on Asthma Control

**Figure 4:**
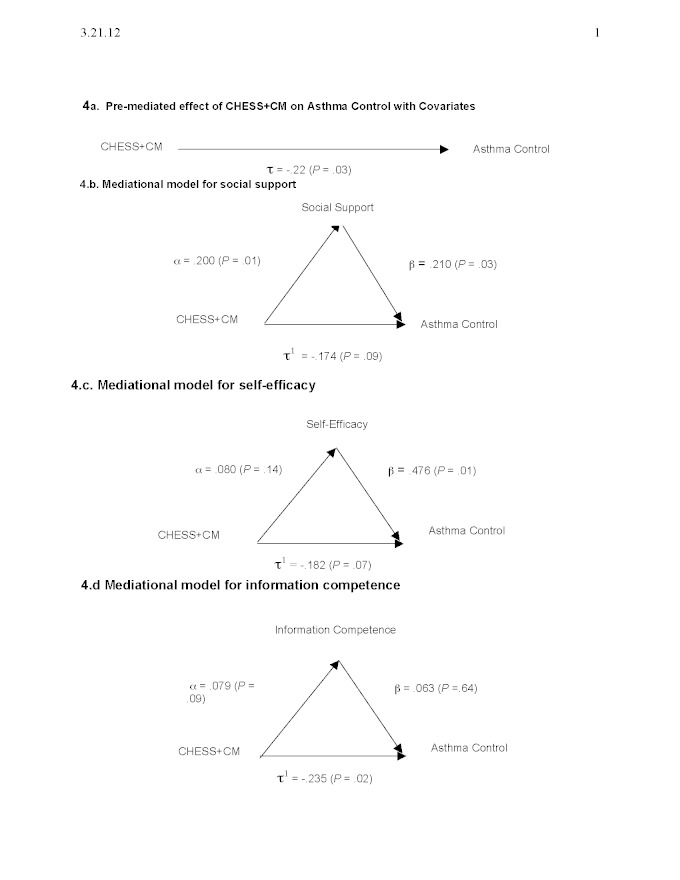
Mediational analyses. CHESS+CM = Comprehensive Health Enhancement Support System plus monthly nurse case management.

Figure 4 shows the prespecified secondary mediational analyses. Part a, which shows the premediated effect of CHESS+CM on the ACQ with all covariates entered into the model, was significant at tau = –.22, *P *= .03. Only Medicaid was a significant covariate. Therefore, we entered three covariates into each mediator model: Medicaid, pretest score from the ACQ, and mediator.

Figure 4 part b shows that CHESS+CM had a significant effect on social support at alpha = .200, *P *= .01, and social support had a significant effect on ACQ at beta = .210, *P *= .03. After mediation, the CHESS+CM effect on ACQ was no longer significant at tau^1 ^= –.174; *P *= .09, as hypothesized.

Figure 4 part c shows that CHESS+CM had a positive but nonsignificant effect on self-efficacy at alpha = .080, *P *= .14. Self-efficacy had a significant effect on ACQ at beta = .476, *P *= .01. After mediation, CHESS+CM no longer had a significant effect on ACQ at tau^1 ^= –.182, *P *= .07.

Figure 4 part d shows that CHESS+CM had a marginally significant positive effect on information competence at alpha = .079, *P *= .09; information competence had a positive but nonsignificant effect on ACQ at beta = .063, *P *= .64. After the mediational analysis, CHESS+CM’s effect on ACQ remained significant at tau^1 ^= –.235; *P *= .02. Information competence, therefore, was not a significant mediator.

In sum, only social support was the only significant mediator for CHESS+CM’s effect on asthma control.

### CHESS Use

**Figure 5:**
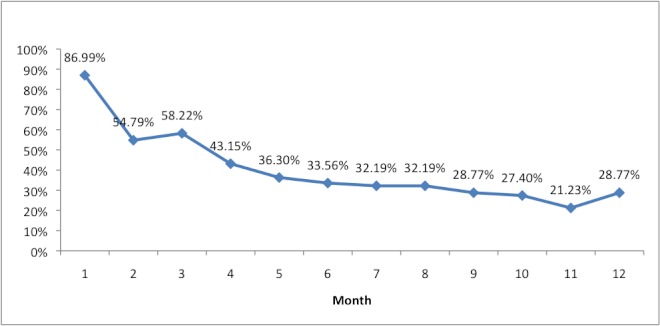
Percentage of active Comprehensive Health Enhancement Support System (CHESS) users by month.

Table 4 and Table 5 present the number of logins, pages viewed, and time spent on the site. Table 4 is based on the total number of potential users (146); Table 5 is based on the number of actual users during each month of access. Figure 5 shows the percentage of potential users that actually used CHESS each month. Figure 5 shows a sharp drop-off from the first to the second month and then small declines in most other months. The same can be said for the extent of use. In the last month, usage rates moved up again, possibly because participants knew that the study was coming to an end. This may have led people to employ their last opportunity to use CHESS or it may reflect users wanting to prepare for the exit interview.

**Table 4:** Logins, pages viewed, and time spent for the 146 potential users.

Measure		Month											
		1	2	3	4	5	6	7	8	9	10	11	12
**Logins**													
	No.	146	146	146	146	146	146	146	146	146	146	146	146
	Mean	10.64	4.15	3.57	2.66	3.12	2.52	2.45	2.60	3.05	2.23	1.40	2.88
	SD	16.72	10.24	7.09	6.91	12.59	9.94	8.33	8.73	11.58	7.25	5.17	10.72
	Minimum	0	0	0	0	0	0	0	0	0	0	0	0
	Maximum	116	89	43	55	126	107	64	73	80	56	46	81
**Pages viewed**													
	No.	146	146	146	146	146	146	146	146	146	146	146	146
	Mean	87.64	29.22	24.12	15.15	13.65	12.08	9.51	8.75	9.56	7.57	6.99	16.47
	SD	111.84	61.29	40.54	32.27	38.75	41.14	22.37	20.08	30.35	22.29	23.66	49.10
	Minimum	0	0	0	0	0	0	0	0	0	0	0	0
	Maximum	649	551	242	231	307	429	167	105	241	193	155	288
**Time (minutes)**													
	No.	146	146	146	146	146	146	146	146	146	146	146	146
	Mean	82.92	25.80	19.52	13.49	12.21	9.21	8.71	9.70	10.25	8.86	8.01	19.50
	SD	131.34	68.01	47.36	48.15	51.45	34.63	34.73	33.03	47.87	46.94	40.23	90.78
	Minimum	0	0	0	0	0	0	0	0	0	0	0	0
	Maximum	760	700	450	533	524	365	354	265	471	517	403	777

**Table 5:** Logins, pages viewed, and time spent for actual users who logged into the Comprehensive Health Enhancement Support System (CHESS) each month.

Measure		Month											
		1	2	3	4	5	6	7	8	9	10	11	12
**Logins**													
	No.	127	80	85	63	53	49	47	47	42	40	31	42
	Mean	12.24	7.58	6.13	6.17	8.60	7.51	7.60	8.09	10.62	8.13	6.61	10.02
	SD	17.38	12.90	8.41	9.47	19.85	16.12	13.37	13.97	19.79	12.10	9.68	18.25
	Minimum	1	1	1	1	1	1	1	1	1	1	1	1
	Maximum	116	89	43	55	126	107	64	73	80	56	46	81
**Pages viewed**													
	No.	127	80	85	63	53	49	47	47	42	40	31	42
	Mean	100.76	53.33	41.44	35.11	37.60	36.00	29.53	27.19	33.24	27.63	32.90	57.24
	SD	114.29	74.79	45.95	41.51	57.18	65.08	31.19	27.55	49.52	35.75	42.72	78.32
	Minimum	0	0	0	0	0	0	0	0	0	0	0	0
	Maximum	649	551	242	231	307	429	167	105	241	193	155	288
**Time (minutes)**													
	No.	127	80	85	63	53	49	47	47	42	40	31	42
	Mean	95.33	47.09	33.53	31.25	33.62	27.45	27.06	30.13	35.62	32.33	37.71	67.79
	SD	136.60	86.45	58.28	69.69	81.53	55.78	57.39	53.02	84.73	86.10	81.64	160.61
	Minimum	0	0	0	0	0	0	0	0	0	0	0	0
	Maximum	760	700	450	533	524	365	354	265	471	517	403	777

## Discussion

### Summary of Primary Outcomes

We report on a randomized controlled trial that integrated an asthma eHealth program called CHESS with case management from a monthly telephone call from an asthma nurse. The study enrolled 305 parent–child dyads and randomized 301 dyads. Half of our sample were African American and had a low income. Despite the digital divide, the intervention showed significant improvement on pediatric asthma control when measured by the ACQ, but not when measured as symptom-free days from asthma diaries. The intervention did not have a significant effect on adherence to asthma controller medications. Pharmacy refill rates improved for both groups, yet self-reported adherence declined in both groups. Social support mediated CHESS+CM’s effect on the ACQ. Despite positive trends, we found no significant effects for self-efficacy or information competence. Still, the study has important implications because of its focus on (1) low-income, high-risk populations (often African American), (2) an integrated system of nurse case management and eHealth, and (3) mediation analysis to identify the mechanisms of the effect of eHealth systems.

### Mediational Effects of Social Support Versus Information

Of the three mediational analyses, social support was the only factor that significantly mediated the CHESS+CM effect on asthma control. CHESS+CM had a marginally significant impact on information competence (*P *= .09), but information competence had no impact on asthma control (*P *= .64). Conversely, CHESS+CM had a nonsignificant but promising trend (*P *= .14) toward improving self-efficacy. The trend was strongly associated with improvements in asthma control (*P *= .01).

These mediational analyses raise an intriguing question about the relative contribution of social support and information in pediatric asthma management and, as a result, the relative effort that should be spent in developing eHealth technologies, at least for asthma. CHESS+CM’s modest improvements in information competence but lack of mediational effect for the ACQ may result from an insensitive measure or inadequate presentation of information about or the nature of asthma care, or may indicate that knowledge may not be as important as motivation. On the other hand, an exploratory study of a Web portal for pediatric diabetes patients and their parents reported that users put great value on obtaining information from the site [45]. It might be that, for this largely low-income and minority sample of parents, communicating specific asthma information by phone and case manager email with trusted, caring case managers is a more effective way to encourage asthma management than is reading information in the CHESS program. While these results may not hold for other chronic diseases, we encourage future researchers to consider the relative effects of social support and health information, and possible ways to deliver health information in a supportive manner. Interventions themselves may benefit from being simplified, leading to a more extensive adoption and use of eHealth systems.

### Challenges of Measuring Medication Adherence

These results confirm other study results that show asthma diary data are unreliable because of lapses in daily record keeping and overreporting of adherence [46]. The self-reported decline in adherence over time in both groups, however, is puzzling and raises questions about whether participation in the study encouraged more candid responses. This might be interpreted as increasing levels of realistic self-evaluation. Missing data about pharmacy refills were high and may stem from administrative errors and a highly mobile Medicaid population. This research might have benefited from the use of electronic medication measurement devices [47], but these are costly, allow for dumping doses, and may augment adherence and thus reduce generalizability [48].

### Limitations

Somewhat surprising was the control group’s significant and sustained improvements in refilling asthma controller medications. Control group placebo effects are widely recognized, but primarily for double-blind medication trials rather than health education interventions. Notably, our control group received a welcoming and thorough nurse-led, hour-long intake that was, for many parents and children, their first asthma-focused clinical appointment. This intake included an assessment of the child’s asthma and parent’s well-being, asthma education as needed, and a warm handoff rather than a referral to follow-up care. Control group participants also received four quarterly mailings with seasonally tailored asthma information, as well as parenting and community resources [49].

In a routine application setting, the case managers would have been employed by the MCO (possibly making our results more optimistic) and the control group would not have received the extensive attention provided in this study (possibly making our results more conservative).

Participants in this eHealth trial were not blinded. This research examined several outcomes of interest (asthma control, symptom-free days, and medication adherence), thus increasing the risk for a type I error. While we did use a Bonferroni correction, we did not use such a correction in the mediation analysis. We do not report analyses comparing users with nonusers of CHESS. Doing so would have increased the length of an already complex paper, shifted attention away from the primary analyses, and introduced substantial biases in research reporting. Finally, eligibility requirements and informed consent limited generalizability of the results. Specifically, we required a level of literacy beyond that possessed by many low-income people.

When this study was initiated, smartphones were just becoming powerful vehicles for change. If we were to do this study over today, we would make several changes. We would use global positioning system tracking to identify when the child entered a prespecified high-risk location, such as a smoker’s home. We would install more reminders to both parents and children. Our social media would have included a service in which parents could exchange tips on how best to promote adherence. We would have added a panic button and services to offer help if a child entered an asthma attack. We would have explored the addition of other sensors, such as a peak flowmeter attached to the smartphone. In a second study, we would compare CHESS alone versus CHESS+CM versus control.

### Comparison with Prior Work

A 2011 Cochrane review of 21 randomized studies found that asthma telehealth care interventions did not show a clinically significant improvement in patients’ quality of life or in the number of emergency department visits, but did show a significant reduction in the number of asthma-related hospitalizations [50]. Most interventions used telephones, and none combined telephone case management with self-guided Web-based education—and none measured the effects on asthma control. Like other pediatric asthma intervention studies with samples, the authors noted that an “active” control group may have reduced the effects on the primary outcomes [51]. Other interventions have improved pediatric asthma outcomes and cognitive learning processes [52,53]. An interactive game that improved children’s asthma knowledge, self-management, and clinical outcomes did not significantly improve self-efficacy but found higher scores correlated with better self-management [53]. A school-based intervention improved asthma self-efficacy, knowledge, and asthma management activities as outcomes, but not asthma control [53]. An eHealth program improved asthma knowledge, which correlated with reduced use of rescue medicine and emergency department visits [54]. However, this is the first study to our knowledge that tested the mediational effects of cognitive learning factors on asthma control.

Similarly to the present study, others have found significant effects from brief asthma educational interventions analogous to our active control condition. A single, brief pediatric asthma educational intervention improved asthma outcomes—at least in the short term [55]. An evaluation of self-management support provided by in-home community health workers compared with an active control of three clinic-based pediatric asthma nurse education sessions found modest significant improvements in symptom-free days [56].

This is one of very few eHealth studies that have shown an effect on pediatric asthma caregivers. This is significant because it confirms the family’s critical role in disease management [57].

### Conclusion and Implications for Further Research

CHESS+CM provided information, social support, and interactive tools to help parents overcome barriers to managing their child’s asthma, and secondarily to help the child participate in his or her asthma management [35]. The present study, however, could not identify whether the relative impact of the CHESS eHealth program or monthly phone conversations with the case manager affected these outcomes. Further analyses are needed to identify the specific effects for the separate components of CHESS and case management for different participant profiles and to provide important clues about how asthma education can be tailored better to meet the complex needs of managing pediatric asthma within the family context.

From an eHealth development perspective, more research is needed into the conditions under which it makes sense to invest heavily in various aspects of disease management [58], such as information versus social support. In the present study, the case manager provided asthma information in a supportive and encouraging manner during the monthly phone call—perhaps conflating the relative contribution of information and social support in improving asthma control. In sum, continuous condition-specific and population-specific research and refinement are needed to develop and implement effective eHealth programs.

Finally, information and communication technologies like the one used here might be cost beneficial in disease management [59]. Efficacy studies of information and communication technologies in chronic disease self-management are promising [60,61]. People with addictions tend to view information and communication technologies favorably [62]. They acknowledge more drug use and psychiatric symptoms online than in face-to-face interviews [63]. Computerized screening and brief interventions have been shown to reduce problem drinking [64-66]. A recent review [67] found positive outcomes in 29 of 32 randomized trials of personal computer interventions offering a single service, such as texting and giving reminders, for various chronic diseases. Randomized controlled trials of smartphone systems are just beginning to appear.

## Multimedia Appendix 1

CONSORT eHealth checklist V1.6 [68].

## Abbreviations

ACQ: Asthma Control Questionnaire
CHESS: Comprehensive Health Enhancement Support System
CHESS+CM: Comprehensive Health Enhancement Support System plus monthly nurse case management
CL: confidence limits
MCO: managed care organization
